# Characterising the effect of *Akirin* knockdown on *Anopheles arabiensis* (Diptera: Culicidae) reproduction and survival, using RNA-mediated interference

**DOI:** 10.1371/journal.pone.0228576

**Published:** 2020-02-12

**Authors:** Blaženka D. Letinić, Yael Dahan-Moss, Lizette L. Koekemoer

**Affiliations:** 1 Wits Research Institute for Malaria, School of Pathology, Faculty of Health Sciences, University of the Witwatersrand, Johannesburg, South Africa; 2 Centre for Emerging Zoonotic and Parasitic Diseases, National Institute for Communicable Diseases of the National Health Laboratory Service, Johannesburg, South Africa; University of Crete, GREECE

## Abstract

*Anopheles arabiensis* is an opportunistic malaria vector that rests and feeds outdoors, circumventing current vector control methods. Furthermore, this vector will readily feed on animal as well as human hosts. Targeting the vector, while feeding on animals, can provide an additional intervention for the current vector control activities. Agricultural animals are regularly vaccinated with recombinant proteins for the control of multiple endo- and ecto-parasitic infestations. The use of a Subolesin-vaccine showed a mark reduction in tick reproductive fitness. The orthologous gene of *Subolesin*, called *Akirin* in insects, might provide a valuable species-specific intervention against outdoor biting *An*. *arabiensis*. However, the biological function of this nuclear protein has not yet been investigated in this mosquito. The effects on *An*. *arabiensis* lifetable parameters were evaluated after *Akirin* was knocked down using commercial small-interfering RNA (siRNA) and *in vitro* transcribed double-stranded RNA (dsRNA). The siRNA mediated interference of *Akirin* significantly reduced fecundity by 17%, fertility by 23% and longevity by 32% when compared to the controls in the female mosquitoes tested. Similarly, dsRNA treatment had a 25% decrease in fecundity, 29% decrease in fertility, and 48% decrease in longevity, when compared to the control treatments. Mosquitoes treated with Akirin dsRNA had a mean survival time of 15-days post-inoculation, which would impact on their ability to transmit malaria parasites. These results strongly suggest that Akirin has a pleiotropic function in *An*. *arabiensis* longevity and reproductive fitness.

## Introduction

Vector-borne diseases cause more than 700,000 deaths annually [1]. The mosquito-borne disease, malaria, is accountable for 60% of these annual deaths [2]. In Africa, malaria is caused by the bite of a *Plasmodium*-infected female mosquito mainly belonging to the *Anopheles funestus* group or the *Anopheles gambiae* complex. The *Anopheles gambiae* complex is comprised of eight species, two of which are minor malaria vectors (*An*. *merus* and *An*. *melas*), while three are major malaria vectors (*An*. *gambiae s*.*s*., *An*. *coluzzii*, and *An*. *arabiensis*) [3, 4]. The main malaria vector in South Africa is *An*. *arabiensis* [5].

In South Africa, malaria primarily occurs in the Limpopo, Mpumalanga and KwaZulu-Natal provinces. While these provinces implement well-coordinated malaria control operations, vector control is based predominantly on the application of indoor residual spraying [6]. *Anopheles arabiensis* mosquitoes are seen as an opportunistic species, as they show variations in resting behaviour (endophilic and exophilic) and feeding behaviour (endophagic, exophagic, anthropophilic and zoophilic) [3] when compared to other *Anopheles* malaria vector species. This makes it practically impossible to eliminate *An*. *arabiensis* using current control methods, contributing to the ongoing transmission of residual malaria in South Africa [5].

This problem is compounded by the fact that insecticide resistance is increasing in the vector population, which is most likely due to increased selection pressure [7]. In South Africa, the *An*. *arabiensis* population has shown resistance to DDT (Dichlorodiphenyltrichloroethane), pyrethroids, and carbamates, especially in northern KwaZulu-Natal [7–9]. This places tremendous pressure on the goal of eliminating malaria from South Africa by 2030 [2], and necessitates the need for the development of additional vector control interventions. However, the identification of additional novel interventions is largely dependent on the identification of new target sites or biological pathways [10].

Akirin is a highly conserved nuclear transcription co-factor [11], that plays a crucial role in innate immunity [12]. The innate immune system is comprised of two distinct signalling pathways, namely the immune deficiency (Imd) pathway and the Toll pathway. Akirin regulates NF-kB dependent transcription in the Imd pathway, as it is required at the level of the transcription factor Relish [12]. RNAi-mediated gene knockdown of *Akirin* in *Drosophila melanogaster* has been shown to impair Imd signal transduction and enhance sensitivity to Gram-negative bacterial infection [12]. *Akirin* knockdown in *An*. *coluzzii* (historically called *An*. *gambiae* M-molecular form [4]) showed an increased susceptibility to *P*. *berghei* infection, supporting its role in mosquito immunity [13, 14].

Akirin also plays a critical role in processes unrelated to immunity, such as embryonic development [12]. Knockdown of its ortholog in ticks, *Subolesin*, resulted in several phenotypic deleterious effects [15, 16]. This included significantly diminished feeding capabilities of engorged ticks, resulting in a notable decline in tick mass [17]. In addition to these phenotypic changes, developmental abnormalities such as tissue damage, failure of nymph metamorphosis, diminished vectorial capacity, reduced survival, and reduction reproductive fitness were also observed [17, 18].

The highly conserved nature of Akirin provides a valuable species-specific intervention against *An*. *arabiensis*. However, the biological function of this nuclear protein has not yet been investigated in this mosquito. The effects on *An*. *arabiensis* lifetable parameters were evaluated, with specific focus on vector fecundity, fertility, and longevity.

## Materials and methods

### Biological material

A laboratory strain of *An*. *arabiensis* mosquitoes (MBN) was used. This strain was colonised in 2002, from wild material collected in Mamfene, northern KwaZulu-Natal, South Africa [8]. It is maintained in the Botha De Meillon Insectary, at the Vector Control Reference Laboratory (VCRL) of the National Institute for Communicable Diseases (NICD) in Johannesburg, under standard insectary conditions of 80% humidity, 25°C, and a 12-hour day/night cycle with 45-minute dusk/dawn transitions [19]. All adult mosquitoes were sustained on a 10% sucrose solution diet. Blood meals were provided to mated female mosquitoes via routine guinea pig blood feeding. Blood feeding protocol and ethics were reviewed and approved by the National Health Laboratory Service (NHLS), Animal Ethics Committee (AEC) (AESC: 1993–047).

Guinea pig colony are maintained at the NHLS animal until issued. Original breeding stock was bought from Harlan UK Labs. Guinea pigs are kept in standard laboratory cages at a temperature range of 21°C (+/- 2°C), with a light/dark cycle of 12 hours. Cages are cleaned three times per week. Guinea pigs are provided with a bed of pine shavings, with added hay/eragrostis, and are provided with commercial rabbit pellets and water ad-lib. Water is supplemented with vitamin C (three times a week). To minimize stress on the animals, staff and routine activities are kept constant in a strict “low noise” environment, preventing unnecessary stress. Animal injections and veterinary procedures were strictly conducted by trained South African Veterinary Council (SAVC) registered staff. Prior to blood feeding, guinea pigs are anaesthetised to prevent distress of the animals.

### RNA interference

RNA interference is a technique that uses exogenous double-stranded RNA (dsRNA) to repress the expression of endogenous messenger RNA (mRNA), subsequently causing loss of gene transcript [20]. Two separate techniques were used to repress the expression of endogenous *Akirin* mRNA in *An*. *arabiensis* mosquitoes. The first technique used commercially available small interfering RNAs (siRNAs), that were synthesised by ThermoFisher using Ambion® *in vivo* chemical modifications to ensure minimal off-target activity. Putative siRNAs were selected based on the *Silencer*® Select algorithm, that was used to analyse the full coding sequence of each gene (See S1 Table for siRNA sequences).

RNAi-mediated knockdown, using Ambion® siRNA, was optimised using Glyceraldehyde 3-phosphate dehydrogenase (GapDH)-specific siRNA as a positive control (ThermoFisher, s541529). *GapDH* was significantly down-regulated by a mean factor of 0.16 (S.E. range 0.11–0.28) (*p* = 0.03) when conducting qPCR. Ambion® *in vivo* custom-designed siRNA was used to target Akirin (ThermoFisher, s541552), while the Ambion® pre-designed Mouse-ß2m siRNA (*in vivo)* was used as a negative control (ThermoFisher, s62844).

The second technique used *in vitro* transcribed dsRNA that was synthesised using RNA that was isolated from 1-day-old untreated female *An*. *arabiensis* mosquitoes. RNA was extracted using Trizol™ (Invitrogen, 15596026) and quantified using a NanoDrop™ spectrophotometer. The integrity of the RNA was assessed using a 1% non-denaturing agarose gel, TBE (Tris/Borate/EDTA) [21]. The gel electrophoresis was conducted at 70V for 30 minutes, to ensure minimal RNA degradation. The isolated RNA was reverse-transcribed into complementary DNA (cDNA) using the QuantiTect® reverse-transcription kit (Qiagen, 205310).

The cDNA was amplified using primers designed to synthesise Akirin dsDNA [13]. The forward primer (^5’^GGTACTTTGGCAGTCGTTGTAGTTGC^3’^) and the reverse primer (^5’^GGTACTCACCTGCTTGAAGGTGAACA^3’^) contained T7 promoters (^5’^TAATACGACTCACTATAG ^3’^) that were appended at the 5’ prime end of the sequence, to allow for *in vitro* transcription to take place. Polymerase chain reaction (PCR) was performed using cDNA (250ng), 1x PCR buffer (10mM Tris-HCl, 5mM KCl), 1mM dNTP’s, 3mM MgCl_2_, 0.4μM forward primer, 0.4μM reverse primer, and 0.1U *Taq* polymerase, which was made up to a final volume of 25μl using nuclease-free water. PCR was conducted by heating the sample to 94°C for 5 minutes, followed by 40 PCR cycles (94°C for 30 seconds, 52°C for 1 minute, 72°C for 1 minute), and a final extension at 72°C for 5 minutes. PCR products were purified using the QIAquick® PCR purification kit (Qiagen, 28104), as per the manufacturer’s specifications. The purified PCR products were quantified using the NanoDrop™ spectrophotometer, after which the integrity and size of the purified PCR products (250ng) were examined on a 1% agarose gel (75 minutes, 110V), TAE (Tris/Acetate/EDTA), prior to *in vitro* transcription.

The MEGAscript® RNAi kit (Invitrogen, AM1626) was used to assemble the transcription reaction, by generating two complementary RNA transcripts. In a 20μl reaction, 1-fold of ATP, CTP GTP, UTP, T7 enzyme mix and reaction buffer was added to 1μg dsDNA template. The sample reactions were incubated at 37°C for 4 hours. Nuclease digestion was performed to digest residual template DNA and ssRNA. Nuclease digestion was conducted by adding 2.5x digestion buffer, 2μl of DNaseI, 2μl of RNase, and 21μl nuclease-free water to the *in vitro* transcribed dsRNA product. This reaction was incubated for 1 hour at 37°C to allow for the template DNA to digest. The digested DNA, free nucleic acids, and residual proteins were removed from the dsRNA using the filter cartridge provided in the MEGAscript® RNAi kit, as specified by the manufacturer. The eluted purified *in vitro* transcribed dsRNA was quantified using the NanoDrop™ spectrophotometer, and the integrity was assessed on a 1% agarose gel, TAE (75 minutes, 110V).

### Inoculation

The Nanoinject II (Drummond, 3–000204) was used to conduct RNAi inoculation on 1-day-old female *An*. *arabiensis* mosquitoes [22]. Cold anesthetised mosquitoes were inoculated with 69nl (3mg/ml) of Akirin dsRNA, Akirin siRNA or Mouse-ß2m siRNA. A subset of cold anesthetised mosquitoes injected with PBS was used as a handling control, while a subset of mosquitoes that were not subjected to inoculation, but had undergone cold anesthetisation, were used as an untreated control. Fatalities as a result of the injection procedure were minimal. However, if a fatality occurred immediately after injection, the mosquito was discarded from the analysis.

Inoculations were conducted using a total of 300 female mosquitoes per each treatment. Fifty mosquitoes were randomly selected from each treatment for quantitative-PCR (qPCR) analysis (10 mosquitoes per replicate, 5 replicates), while 150 female mosquitoes were randomly selected for longevity analysis (30 mosquitoes per replicate, 5 replicates). The remaining 100 female mosquitoes from each treatment were used to analyse vector fertility and fecundity (20 mosquitoes per replicate, 5 replicates). All treatments were carried out in 32.5cm^3^ insect rearing cages (BugDorm®, 211476).

### Quantitative-PCR

Previous *Akirin* knockdown studies showed a 16–40% decrease in Akirin expression in the midgut of the mosquito, while the remaining tissues showed a 25–65% decrease in Akirin expression [13]. For this reason, RNA was extracted from whole mosquito samples three days post-RNAi inoculation. RNA was reverse-transcribed into cDNA using the QuantiTect® reverse-transcription kit. Quantitative-PCR was conducted using 1x iQ™ SYBR® Green Supermix (Bio-Rad, 170–8887), 0.48μM forward primer, 0.48μM reverse primer, and 100ng cDNA template, which was made up to a final volume of 25μl using nuclease-free water. All samples were amplified using the C1000^™^ thermal-cycler, Bio-Rad CFX96 Touch^™^ real-time PCR detection system. PCR amplification was performed by preheating the reaction to 94°C for 2 minutes, followed by 40 PCR cycles (94°C for 30 seconds, 62°C for 30 seconds, 72°C for 40 seconds), and a final PCR extension of 10 minutes at 72°C.

The relative expression ratios of the *Akirin* knockdown samples were measured in comparison to the *Mouse-ß2m* knockdown samples using REST^©^ analysis (relative expression software tool). The relative expression ratios of *Mouse-ß2m* knockdown samples were also compared to the PBS and untreated samples in each treatment, to ensure that the results being observed were gene-specific. Gene expression was normalised using *RPS7* and *RPS26* as reference genes (See S2 Table for primer sequences).

### Fecundity and fertility

Mosquito fecundity and fertility were analysed post-inoculation using a total of 100 female mosquitoes per treatment (20 mosquitoes per replicate, 5 replicates). The female mosquitoes were placed in cages with untreated 2-day-old male mosquitoes (20 mosquitoes per cage) for a period of 4-days to allow mating to take place, after which the male mosquitoes were removed from the cages. Two subsequent blood meals were offered to the mated females over five days [23]. After receiving the second blood meal, each female was transferred into a separate paper cup (8cm diameter, 9cm height), with a net fastened over the top of the cup using an elastic band. Approximately 20ml of distilled water was poured into each cup. Mosquitoes were maintained on a 10% sucrose solution until oviposition had taken place. Vector fecundity was determined by calculating the mean number of eggs laid per female. The hatch rate was monitored over 10-days. Eggs which did not hatch 10-days post oviposition were deemed infertile. Vector fertility was determined by calculating the mean percentage of hatchlings per female.

### Longevity

Longevity was assessed to determine whether *Akirin* knockdown affected *An*. *arabiensis* survival. Longevity was evaluated using a total of 150 female mosquitoes per treatment (30 mosquitoes per replicate, 5 replicates). The rate of survival was monitored until 100% mortality was reached for all five treatments (Akirin dsRNA, Akirin siRNA, Mouse-ß2m siRNA, PBS, and untreated). A Kaplan-Meier survival curve was constructed using Statistix 10, where the probability of survival for each treatment was displayed as horizontal lines on the curve. The vertical line’s distance between the horizontal lines illustrated the change in cumulative probability, which changed when a death occurred [24].

### Statistical analysis

All statistical analysis was performed at a 95% confidence interval. A one-way analysis of variance (ANOVA) and a Tukey HSD post-hoc test were used to compare the mean fecundity and fertility between the treatments. The Log-rank test was used to determine whether there was a statistically significant difference in vector longevity between the various treatments.

## Results

### Quantitative-PCR

**Fig 1:**
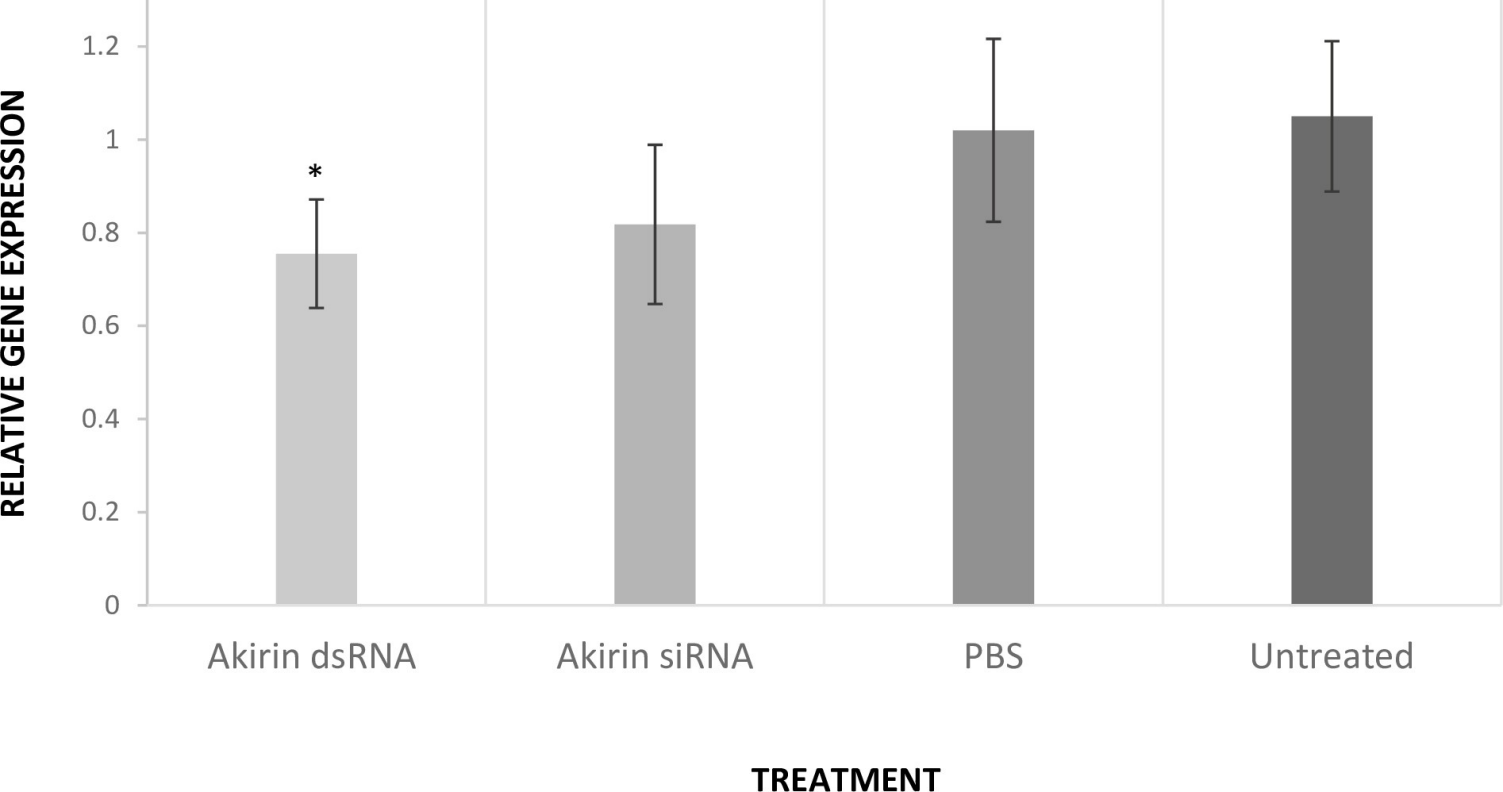
The change in *Akirin* relative expression assessed 3-days post-RNAi treatment. The relative expression of *Akirin* was significantly downregulated by a mean factor of 0.76 (*p* = 0.02), when comparing the expression ratios between the Akirin dsRNA treated samples and the negative control samples. The relative expression of *Akirin* remained unchanged when comparing the expression ratios of the negative control samples (Mouse-ß2m siRNA) to the PBS treated samples (*p* = 0.78) and untreated samples (*p* = 0.83).

Samples treated with Akirin-specific dsRNA showed a significant reduction in *Akirin* expression, by a mean factor of 0.76 (S.E. range is 0.65–0.88) (*p* = 0.02), when compared to the negative control samples (Fig 1). As expected, no significant change was detected amongst the control treatments, confirming that the change in relative expression was gene-specific. The change in *Akirin* expression was also assessed in the samples treated with Akirin-specific siRNA. Although the Akirin-specific siRNA treated samples showed a reduction in *Akirin* expression, by a mean factor of 0.82 (S.E. range is 0.65–0.99) (*p* = 0.06) when compared to the negative control samples, the change in expression was not statistically significant at a 95% confidence interval.

### Fecundity and fertility

**Fig 2:**
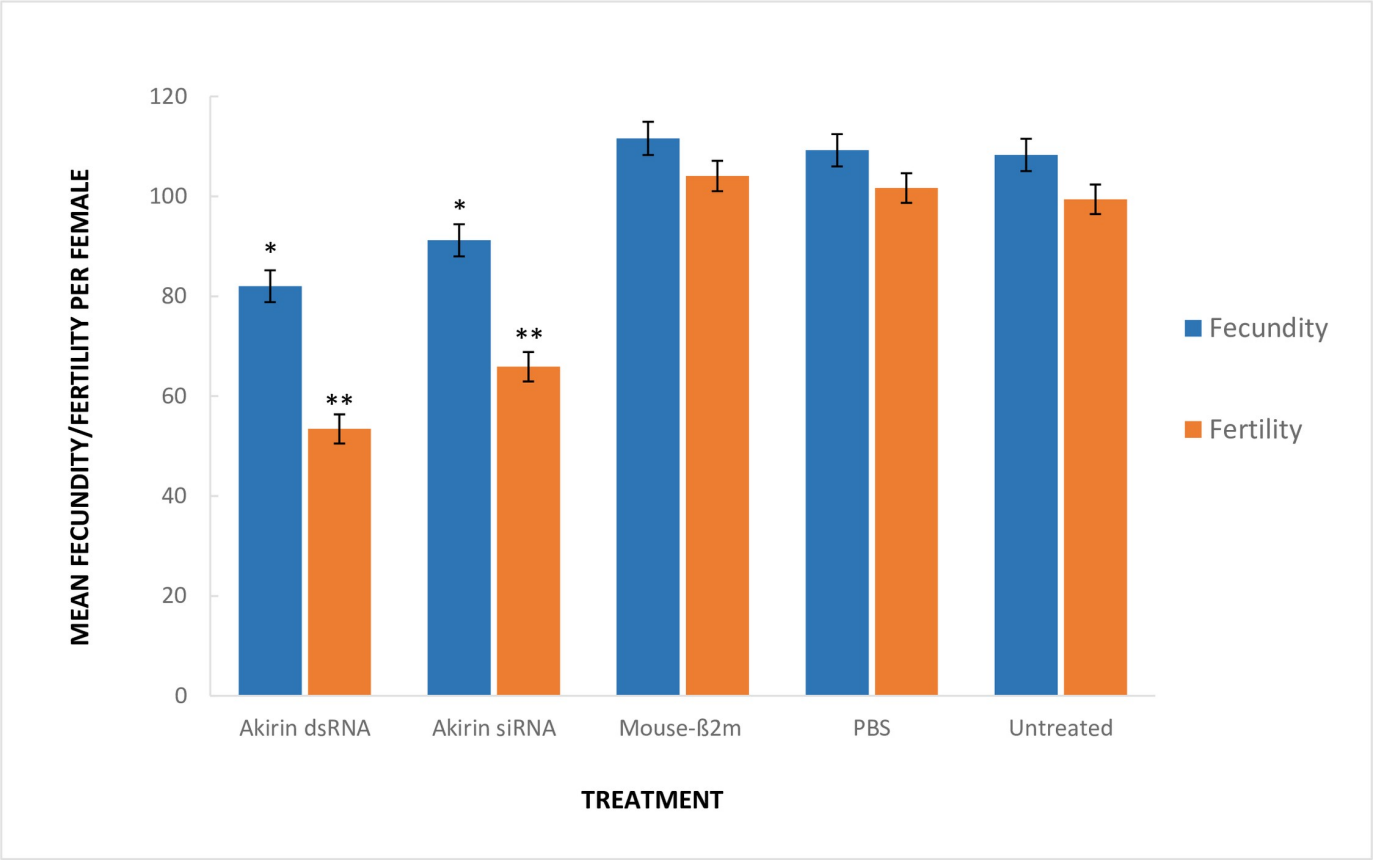
Mean vector fecundity (blue) and fertility (orange) per treatment. *Akirin* knockdown mosquitoes had a reduced mean fecundity by 17–25% (one-way ANOVA: *p*<0.01, F = 16.5, DF = 4), and a reduced mean fertility by 23–29% (one-way ANOVA: *p*<0.01, F = 63.4, DF = 4), when compared to the control treatments. This was a 19–26% decrease in hatch rate percentage. No significant change in fecundity/fertility was observed between the control treatments. The asterisks indicate a significant change from the control treatments.

Mosquito fecundity and fertility were assessed between the various treatments, to determine whether *Akirin* knockdown had an impact on *An*. *arabiensis* oviposition (Fig 2). Mosquitoes treated with Akirin-specific siRNA and Akirin-specific dsRNA had a 1.3-fold (17%) and 1.4-fold (25%) decrease in fecundity respectively, when compared to the control treatments (one-way ANOVA: *p*<0.01, F = 16.5, DF = 4). The control treatments had a mean fecundity of approximately 110 eggs laid per female mosquito, while those treated with Akirin-specific siRNA and Akirin-specific dsRNA had mean fecundity of 91 and 82 eggs laid per female respectively. Mosquitoes treated with Akirin-specific siRNA and Akirin-specific dsRNA had a further 1.2-fold (19%) and 1.4-fold (26%) decrease in fertility respectively, when compared to the control treatments (one-way ANOVA: *p*<0.01, F = 63.4, DF = 4). The control treatments had a mean hatch rate of 92%, while those treated with Akirin-specific siRNA and Akirin-specific dsRNA had a mean hatch rate of 72% and 65% respectively.

### Longevity

**Fig 3:**
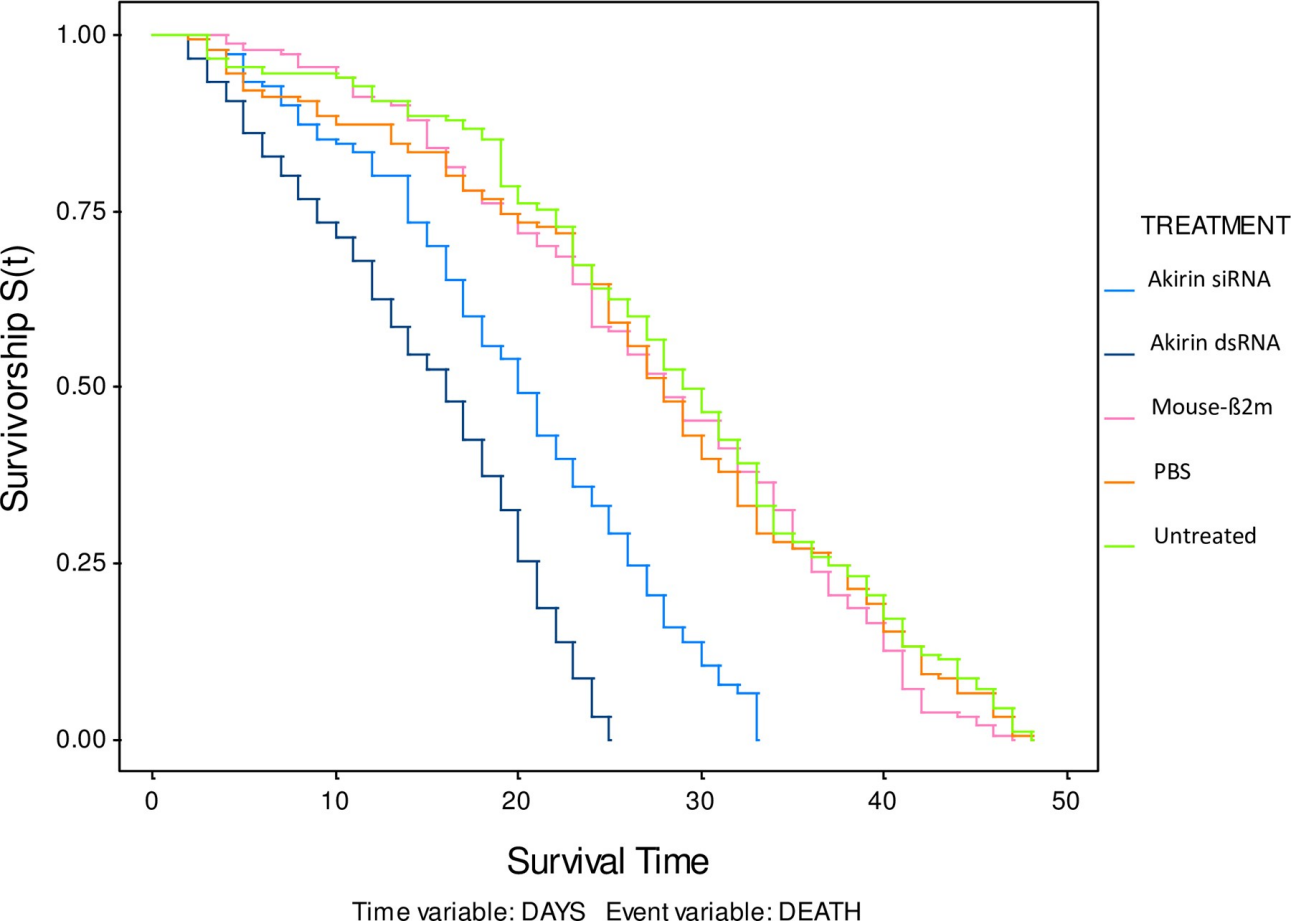
Kaplan-Meier survival analysis of Akirin knockdown (blue), Mouse-ß2m (pink), PBS (orange) and untreated (green) mosquitoes. Female mosquitoes treated with Akirin siRNA and Akirin dsRNA reached 100% mortality 15 and 23-days (respectively) before the control treatments, which conveyed a 32–48% decrease in vector longevity (Log-rank: χ^2^ = 154, *p*<0.01, DF = 4).

The results of the longevity experiments are shown in Fig 3. The control treatments had a mean survival time of 28-days, while the mosquitoes treated with Akirin siRNA and Akirin dsRNA had a mean survival time of 20-days and 15-days post-inoculation respectively. At 15-days post-inoculation, mosquitoes treated with Akirin siRNA had a mortality of 35%, while the mosquitoes treated with Akirin dsRNA had a mortality of 52%. This was significantly higher than the controls (Log-rank: χ^2^ = 78, *p*<0.01, DF = 4), as the mosquitoes treated with Mouse-ß2m siRNA and PBS had a mortality of 20%, and the untreated mosquitoes had a mortality of 12%. Mosquitoes treated with Akirin siRNA and Akirin dsRNA reached 100% mortality 33-days and 25-days post-inoculation respectively, while the control treatments reached 100% mortality after 47-days. When compared to the control treatments, the mosquitoes treated with Akirin siRNA had a 32% decrease in longevity overall, while the mosquitoes treated with Akirin dsRNA had a 48% decrease in longevity in total (Log-rank: χ^2^ = 154, *p*<0.01, DF = 4).

## Discussion

Characterising the effect of *Akirin* knockdown on *An*. *arabiensis* provided insight into its pleiotropic function in insects, and its value as a species-specific intervention against *An*. *arabiensis*. Two different knockdown molecules (dsRNA and siRNA) were used to repress the expression of endogenous *Akirin* mRNA in *An*. *arabiensis* mosquitoes. Regardless of the molecule used for repression, a statistically significant change in vector fecundity, fertility, and longevity was observed. This was consistent with the results obtained when *Akirin* was downregulated in *An*. *coluzzii*, where fecundity was reduced by 52% and survival by 14% [13]. Although Akirin is known to be involved in various other physiological and developmental pathways, no other visible phenotypic differences were observed between the *Akirin* knockdown mosquitoes and the controls.

The *in vitro* transcribed exogenous dsRNA was, however, more efficient in repressing *Akirin* expression in *An*. *arabiensis* than the commercially available exogenous siRNA. This was due to improved absorption uptake of the dsRNA [25], as well as asymmetry in the assembly of the RNAi enzyme complex [26]. Although less efficient, deleterious effects were still apparent when assessing the lifetable parameters in the mosquitoes treated with Akirin-specific siRNA. This suggested that *Akirin* expression could have recovered shortly after being repressed however, its downregulation still had downstream physiological effects in *An*. *arabiensis* [27].

Akirin is required in the Imd pathway [14]. Upon immune challenge, Akirin binds to BAP60, which is a component of the Brahma (SWI/SNF) ATP-dependent chromatin-remodelling complex [12]. The Akirin-BAP60 complex then binds to Relish, which is NF-kB transcription factor, forming a link between Relish and the BAP complex on the promoter of a subset of NF-kB target genes [12, 15, 28]. This link allows for efficient anti-microbial peptide synthesis [29]. The downregulation of Akirin impairs the gene expression of several antimicrobial peptide-coding genes, weakening the innate immune defence required for survival [29].

Mosquitoes treated with Akirin-specific dsRNA had a 54% reduction in mean survival time in relation to the controls. The control treatments had a mean survival time of 28-days, while the mosquitoes treated with Akirin-specific dsRNA had a mean survival time 15-days post-inoculation. This is a significant finding since *Anopheles* mosquitoes are able to transmit malaria parasites 14-days post gametocyte infection [30]. This provides the premise for future vaccine development using recombinant *An*. *arabiensis* Akirin as a potential species-specific antigen for the control of *An*. *arabiensis*.

The recombinant Akirin vaccine would be administered to various reservoir hosts, to elicit an antibody response against the nonself epitopes [13, 31]. Mosquitoes feeding on the vaccinated hosts would ingest antibodies specific to the target antigen. Once ingested, the antibodies would be transported across the gut barrier into the haemolymph, entering the mosquito’s cells [18, 32]. The antibodies would interact with cytosolic Akirin preventing translocation to the nucleus. This would inhibit Akirin from exerting its regulatory function, causing deleterious effects within the vector [13, 18, 31, 32].

Recombinant Akirin antigens have previously been tested against several Culicidae species, with varying results. The ingestion of anti-Akirin antibodies, from mice vaccinated with recombinant *Aedes albopictus* Akirin, caused a 29% decrease in longevity of the European malaria vector, *An*. *atroparvus* [33]. Similarly, vector longevity was also reduced in *Ae*. *caspius* (29%), *Ae*. *albopictus* (17%) and *Culex pipiens* (11%) [33, 34]. However, vector longevity was not affected when *An*. *coluzzii* and *Ae*. *aegypti* ingested recombinant *Ae*. *albopictus* anti-Akirin antibodies [13, 33]. These differences were attributed to the physiological difference between the various species [34], which may be overcome by using a species-specific antigen approach.

The data presented here, as well as the opportunistic feeding behaviour characteristic of *An*. *arabiensis* females, provides the impetus to evaluate the use of recombinant Akirin vaccines in this major African malaria vector.

## Conclusions

*Akirin* knockdown in *An*. *arabiensis* female mosquitoes significantly reduced longevity, fecundity and fertility, suggesting that Akirin has a pleiotropic function in *An*. *arabiensis* survivorship and reproductive fitness.

## Supporting information

S1 Table siRNA sequences used to conduct RNAi. (DOCX)

S2 Table Quantitative-PCR primer sequence. (DOCX)
