# Supplementary material for: Characterising the effect of Akirin knockdown on Anopheles arabiensis (Diptera: Culicidae) reproduction and survival, using RNA-mediated interference
Source: PLoS One. 2020 Feb 12;15(2):e0228576. doi: 10.1371/journal.pone.0228576 (PMC7015393; doi:10.1371/journal.pone.0228576)
Supplement: S1 Table — (DOCX) [file pone.0228576.s001.docx]

**Table 1.** **siRNA sequences used to conduct RNAi**

| **Gene** | **Accession Number** | **Sense Primer** | **Anti-Sense Primer** | **Amplicon Size (bp)** | **Position (bp)** |
| --- | --- | --- | --- | --- | --- |
| Akirin | AARA009142 | ^5’^AUAUCACGGAGGAAAUCAAtt^3’^ | ^5’^UUGAUUUCCUCCGUGAUAUtt^3’^ | 19 | 991-1009 |
| Mouse-ß2m | NM_009735 | ^5’^GCCUGUAUGCUAUCCAGAAtt^3’^ | ^5’^UUCUGGAUAGCAUACAGGCcg^3’^ | 19 | 101- 119 |
| GapDH | AARA011366 | ^5’^AGAUCGGAAUUAACGGAUUtt^3’^ | ^5’^AAUCCGUUAAUUCCGAUCUtc^3’^ | 19 | 8-26 |
