# Supplementary material for: Characterising the effect of Akirin knockdown on Anopheles arabiensis (Diptera: Culicidae) reproduction and survival, using RNA-mediated interference
Source: PLoS One. 2020 Feb 12;15(2):e0228576. doi: 10.1371/journal.pone.0228576 (PMC7015393; doi:10.1371/journal.pone.0228576)
Supplement: S2 Table — (DOCX) [file pone.0228576.s002.docx]

| **Gene** | **Accession Number** | **Sense Primer** | **Anti-Sense Primer** | **Amplicon Size (bp)** | **Position (bp)** |
| --- | --- | --- | --- | --- | --- |
| Akirin | AARA009142 | ^5’^ AGACAGCCCTCCTAGCAT ^3’^ | ^5’^ GTTCGGCTAGTTTAGTGGTC ^3’^ | 149 | 1118-1266 |
| GapDH | AARA011366 | ^5’^GACTGCCACTCGTCCATC^3’^ | ^5’^CCTTGGTCTGCATGTACTTG^3^ | 139 | 856-994 |
| RPS7 | AGAP010592 | ^5’^ AGAACCAGCAGACCACCATC ^3’^ | ^5’^ GCTGCAAACTTCGGCTATTC ^3’^ | 149 | 547-695 |
| RPS26 | AARA003197 | ^5’^ GATAAGGCGATCAAGAAGTTCG ^3^ | ^5’^ TACGGACAACCTTCGAGTGG ^3’^ | 160 | 167-325 |

**Table 2. Quantitative-PCR Primer Sequences**
